# Cannabis Extract Effects on Metabolic Parameters and Gut Microbiota Composition in a Mice Model of NAFLD and Obesity

**DOI:** 10.1155/2022/7964018

**Published:** 2022-06-26

**Authors:** Gil Zandani, Sarit Anavi-Cohen, Tal Assa-Glazer, Jonathan Gorelick, Abraham Nyska, Noa Sela, Nirit Bernstein, Zecharia Madar

**Affiliations:** ^1^The Faculty of Agriculture, Food and Environment, The Hebrew University of Jerusalem, Rehovot, Israel; ^2^Peres Academic Center, Rehovot, Israel; ^3^Eastern Regional R&D Center, Kiryat Arba, Israel; ^4^Sackler School of Medicine, Tel Aviv University, Tel Aviv, Israel; ^5^Department of Plant Pathology and Weed Research, Volcani Center, Rishon LeZion, Israel; ^6^Institute of Soil, Water and Environmental Sciences, Volcani Center, Rishon LeZion, Israel

## Abstract

Nonalcoholic fatty liver disease (NAFLD) is a major cause of chronic liver abnormalities and has been linked with metabolic syndrome hallmarks. Unfortunately, current treatments are limited. This work aimed to elucidate the effects of three cannabis extracts on metabolic alteration and gut microbiota composition in a mouse model of NAFLD and obesity. Male mice were fed with a high-fat diet (HFD) for 12 weeks. Following the establishment of obesity, the HFD-fed group was subdivided into HFD or HFD that was supplemented with one of three cannabis extracts (CN1, CN2, and CN6) for additional 8 weeks. Metabolic parameters together with intestinal microbiota composition were evaluated. Except for several minor changes in gene expression, no profound metabolic effect was found due to cannabis extracts addition. Nevertheless, marked changes were observed in gut microbiota diversity and composition, with CN1 and CN6 exhibiting microbial abundance patterns that are associated with more beneficial outcomes. Taken together, specific cannabis extracts' addition to an HFD results in more favorable modifications in gut microbiota. Although no marked metabolic effect was disclosed, longer treatments duration and/or higher extracts concentrations may be needed. More research is required to ascertain this conjecture and to establish the influence of various cannabis extracts on host health in general and NAFLD in particular.

## 1. Introduction

Nonalcoholic fatty liver disease (NAFLD) is currently the most common liver disease in the Western world, affecting ∼8–45% of the population, globally. NAFLD comprises a wide spectrum of liver pathologies ranging from simple steatosis, which is generally considered benign, through steatohepatitis, cirrhosis, and liver hepatocellular carcinoma [1]. As indicated by its name, the establishment of hepatic fat accumulation and subsequently further perturbations under this state evolve in the absence of excessive alcohol consumption, autoimmune, infectious, or other established liver diseases [2]. NAFLD is ranked as the second most common cause of hepatocellular carcinoma [3] and is implicated as a risk factor for the development of other metabolic-related pathologies, such as cardiovascular diseases, type 2 diabetes, and chronic kidney disease [4]. This emphasizes the relevancy of this disease as a major health problem worldwide and further calls for the development of new, suitable preventative and clinical treatments. Unfortunately, current treatments are restricted, which poses a major challenge for national health services. In the lack of approved pharmaceutical treatment [1], presently available therapeutic approaches focus predominantly on lifestyle modification, diet, and exercise interventions, thus aiming mainly at controlling body weight (BW) and metabolic syndrome-related cardiovascular risk factors [5]. In advanced stages of NAFLD, high genetic risk, or in the presence of diabetes, intensified lifestyle intervention may be accompanied by secondary pharmacological treatment if necessary.

Throughout human history, plants have been used as a source of medications. The species *Cannabis* sativa L. includes three subspecies*: Cannabis sativa, Cannabis indica,* and *Cannabis ruderalis*. The unique therapeutic effects of cannabis are hypothesized to be achieved through a complex synergy between the multiple phytocannabinoids and many other secondary compounds of the plant, including terpenes and flavonoids [6, 7]. Cannabinoids are the subject of ongoing active research in the field of natural pharmaceutical agents, with evidence implying their potential effectiveness for the treatment of inflammation, cancer, and cardiovascular disorders [8–10]. Furthermore, cannabis usage is associated with a reduced risk for metabolic diseases such as obesity [11], diabetes [12, 13], and NAFLD [14, 15].

The interconnection between gut microbiota and the liver in general and NAFLD specifically is of great interest. Dysbiosis has been implicated as a substantial environmental factor involved in the pathogenesis of NAFLD [16]. In patients with NAFLD, the microbiome abundance [17] and community structure are altered [18, 19], suggesting that the gut microbial population is important in the prevention and treatment of NAFLD. However, the effect of cannabis plant extracts on the pathogenesis and progression of NAFLD is not yet clear. Thus, this investigation aims to examine the potential of different cannabis strains that differ in their phytocannabinoids profile as a supplement in food on mice induced NAFLD model and evaluate changes in metabolic parameters, liver fat accumulation, and gut microbiome composition.

## 2. Materials and Methods

### 2.1. Cannabis Plant Extract Preparation, Decarboxylation, and Analysis

Three varieties of *Cannabis* plants were selected for the study based on the composition of the cannabinoids delta-9-tetrahydrocannabinol (THC) and cannabidiol (CBD) as previously described [20]. Briefly, inflorescence from three strains was dried and cured over 4 weeks under 19°C and 40% reactive humidity. Ten grams of dry material were sampled from 10 different inflorescences for each strain, from 10 replicated plants per strain (CN1, CN2, and CN6) and ground into powder. Three grams of ground material were mixed with 30 ml ethanol for 60 min and centrifuged. The supernatants were filtered, dried, and stored at -20°C until further use. In order to convert the cannabinoids from their natural acidic state to their active neutral form, decarboxylation at 110°C for 60 min was performed. Dried extracts were heated, redissolved in ethanol, and chemical analysis of the cannabinoids content of the decarboxylated extracts was performed. 10 µl from each decarboxylated extract was syringe filtered (0.22 µm), diluted in methanol (1:100), and injected into a Waters Alliance 2695 Separation Module with a Waters 996 Photodiode Array Detector together with a Micromass Quattro Micro Triple Quadrupole Mass Spectrometer. Chromatographic separation was achieved using a PhenomenexKinetex C18 column (2.6 μm, 150 mm×3 mm i.d.) with guard column and a binaryA/B gradient (solvent A: water with 0.1% formic acid, and solvent B: MeOH with0.1% formic acid). Initial conditions were 65% B for 10 min, raised to 95% B over the next 20 min, held at 95% B for 15 min, decreased to 65% B over the next 5 min, and held at 65% B for 10 min for re-equilibration of the system. The flow rate was 0.2 mL/min and the column temperature was 30°C. MS acquisition was carried out in the ESI positive ionization: capillary voltage -3.5kV, cone voltage - 45V, extractor voltage – 3V, RF lens - 0.2V, source temperature -120°C, desolvation temperature - 350 °C, and nitrogen flow rate of 700 L/h. For each compound, serial dilutions were performed using standard solutions of the selected cannabinoids, obtained from Sigma-Aldrich, and calibrations curves generated for concentrations from 0.5-100 µg/ml using SIR of the molecular ion[M+H]. Quantification of cannabinoids in the extracts was performed based on the external calibration curves. The plant extract from the CN1 strain was rich in CBD (5 mg/ml), the plant extract from CN2 rich in THC (5 mg/ml while the plant extract from CN6 contained comparable concentrations of both phytocannabinoids (2.5:2.5 mg/ml).

### 2.2. Experimental Animals and Diets

The experiment protocols and procurers were performed within the guidelines of the Authority for Biological and Biomedical Models and approved by the Institutional Animal Care Ethics Committee, both of the Hebrew University of Jerusalem (AG-14922-2). 48 male C57BL/6 J mice, 4-5 weeks old, were purchased from Harlan Laboratories (Jerusalem, Israel). Following the acclimatization period, mice were randomly divided into two experimental groups: (1) mice fed a normal diet (ND, *n* = 8) and (2) mice fed a high-fat diet (60% kcal, HFD, *n* = 40) for 12 weeks. At week 13 of the experiment, HFD mice were divided into 4 groups receiving the following diets for 8 weeks: (1) mice continuing to receive HFD (HFD, *n* = 8), (2) mice fed HFD supplemented with 5 mg/kg·BW of CN1 plant extract (HFD + CN1, *n* = 10), (3) mice fed HFD supplemented with 5 mg/kg·BW of CN2 plant extract (HFD + CN2, *n* = 10), and (4) mice fed HFD supplemented with 5 mg/kg·BW of CN6 plant extract (HFD + CN6, *n* = 10). The amount (mg) of active ingredient was calculated and refers to the most common cannabinoid in each cannabis extract strain, which was incorporated in mice food. Mice were housed under a controlled environment (12/12 h light/dark cycle, 18–24°C, humidity 60%) and provided with *ad libitum* access to food and water. The diet compositions are presented in Table 1. BWs and food intake were recorded weekly.

### 2.3. Oral Glucose Tolerance Test (OGTT)

Glucose-loading tests were conducted at weeks 11 and 19 of the experimental period. Prior to the OGTT, the mice fasted for 12 hours and were given D-glucose (3 g/kg BW) via gavage. Blood was drawn from the tail tip at 0, 30, 60, and 120 min after the glucose loading was used to monitor glucose levels by a glucometer (Optimum Xceed).

### 2.4. Animal Sacrifice and Organ Collection

At the end of the experiment, mice fasted overnight and their BW was recorded thereafter. Mice were sacrificed in a random order by isoflurane (Minard Inc., USA) anesthesia. Blood was collected from the vena cava, centrifuged at 8,000 rpm at 4°C for 10 min, and stored at −80°C. Adipose tissue was removed, weighed, placed in liquid nitrogen, and stored at −80°C. Liver tissue was collected and weighed. A small sample from the right liver lobe was placed in 4% formaldehyde, and the remaining liver tissue was minced in liquid nitrogen and stored at −80°C. The ceca were separated from the large intestines and their contents were collected for microbiota analysis.

### 2.5. Biochemical Analysis of Serum Parameters

Analysis of serum alkaline phosphatase (ALP), serum alanine aminotransferase (ALT), and serum aspartate aminotransferase (AST) were measured by an automated clinical chemistry analyzer along with total cholesterol, high-density lipoprotein (HDL), and total triglycerides (TG) (American Laboratories Ltd., Herzliya, Israel). Total lipid was extracted from livers using the Folch method [21].

### 2.6. Liver Histology Examination and Grading

Histological slides were prepared and stained with hematoxylin and eosin (H&E) by Patholab (Rehovot, Israel) as described in [22]. Histopathological examinations were carry out by Dr. Abraham Nyska, DVM, Dipl. ECVP, Fellow IATP, board-certified in toxicologic pathology—https://ebvs.eu/colleges/ECVP/members/prof-abraham-nyska. Examination and scoring of the histopathological changes were done by the study pathologist using semiquantitative grading of five grades (0–4), according to the severity of the changes according to a generic grading criterium: 0 = no lesion; 1 = minimal change; 2 = mild change; 3 = moderate change; and 4 = marked change.

### 2.7. Western Blot Analysis

Liver tissue lysates were prepared using lysis buffer as previously described [23]. Aliquots of protein were then subjected to western blot analysis. Ponceau S (Sigma) staining was used to verify equal loading and transfer [24, 25]. Blots were respectively incubated with dilutions of primary antibodies: anti-rabbit AMPK, anti-pAMPK (Thr-172), anti-cannabinoid receptor 1 (CB1), and anti-cannabinoid receptor (CB2) (Abcam) at 4° overnight. After several washes, the membranes were incubated with a secondary goat antibody (Jackson Immuno-Research Laboratories, West Grove, PA, USA). The immune reaction was detected by enhanced chemiluminescence, with bands being quantified by densitometry and expressed as arbitrary units.

### 2.8. Quantitative Real-Time PCR

Total RNA was isolated from liver tissues using Tri-Reagent (Sigma-Aldrich, Rehovot, Israel) method, according to the manufacturer's protocol. Complementary DNA was prepared with the High-Capacity cDNA Reverse Transcription Kit (Quanta BioSciences, Gaithersburg, MD, USA). Real-time polymerase chain reaction (PCR) was performed using the 7300 Real-Time PCR System (Applied Biosystems, Foster City, CA, USA), using specific primers as follows: glucose 6-phosphatase (G6pase); peroxisome proliferator-activated receptor alpha (PPAR*α*); a cluster of differentiation 36 (CD36); peroxisome proliferator-activated receptor gamma coactivator 1-alpha (PGC-1*α*); and phosphoenolpyruvate carboxykinase (PEPCK). Fold change in gene expression was determined by normalizing to 18S mRNA. The primer sequences are listed in Table 2.

### 2.9. Gut Microbiota Analysis

The effects of each diet on the gut microbiome population were examined with the analysis of the prokaryotic 16S ribosomal RNA gene (16S rRNA) as previously described [26]. Sequences with 97% similarity were assigned to the same operational taxonomic units (OTU). OTUs of representative sequences at a similarity of 97% and their relative abundances were used to calculate and analyze rarefaction curves and bacterial abundance at all taxonomical levels. Bacterial richness and diversity within samples were classified by *α*-diversity indexes.

### 2.10. Statistical Analysis

Results are presented as mean ± standard error of mean (SEM). Data were analyzed by the JMP 14 Pro software suites (SAS Institute, Cary, NC, USA). Comparisons between groups were made by one-way analysis of variance (ANOVA) followed by a Tukey–Kramer test or by an unpaired two-tailed Student's *t*-test. Statistical significance was defined at *p* < 0.05.

## 3. Results

### 3.1. BW, Food Intake, Tissues Weight, and Liver Steatosis

**Figure 1:**
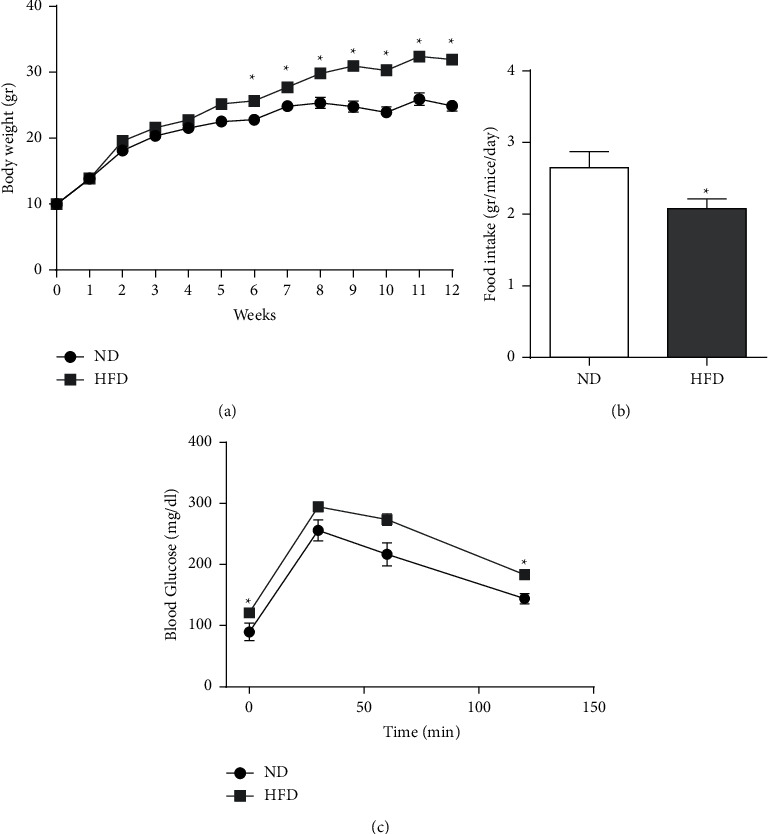
Effects on BW, food intake, tissues weight, and glucose tolerance. Male C57BL/6 J mice were fed with ND or HFD for 12 weeks. (a) BW over experiment duration. (b) Average food intake per mice per day over the experiment duration. (c) Glucose levels between 0 to 120 min during OGTT. All values are expressed as mean ± SEM (*n* = 8–40). Columns and graphs marked with ^*∗*^ are significantly different from ND (*p* < 0.05).

**Figure 2:**
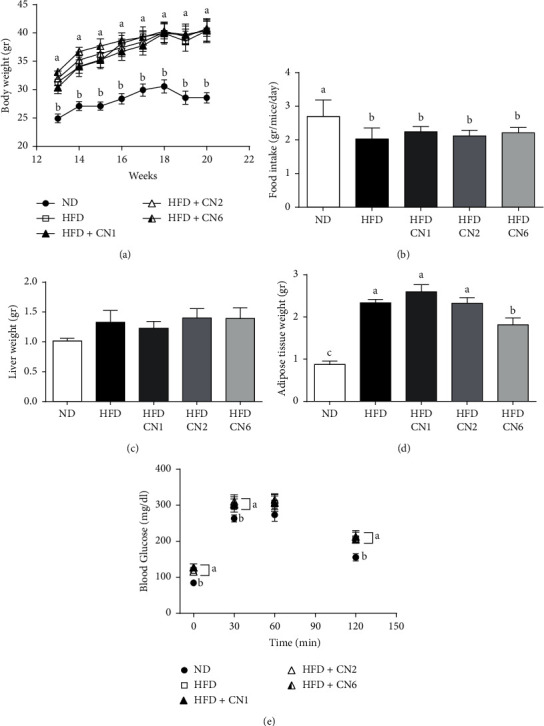
Effect on BW gain, food intake, liver, and adipose tissues weight, and glucose tolerance. Male C57BL/6 J mice fed a high-fat diet supplemented with CN1 or CN2 or CN6 extracts 5 mg/kg BW for 8 weeks. (a) Changes of BW throughout cannabis extracts supplementation. (b) Average food intake per mice per day over the cannabis extract supplementation period; (c) liver weight at sacrifice; (d) adipose tissue weight at sacrifice. (e) Glucose levels between 0 and 120 min during oral glucose tolerance test. All values are expressed as mean ± SEM (*n* = 8–10). Columns marked with different letters are significantly different (*p* < 0.05).

**Figure 3:**
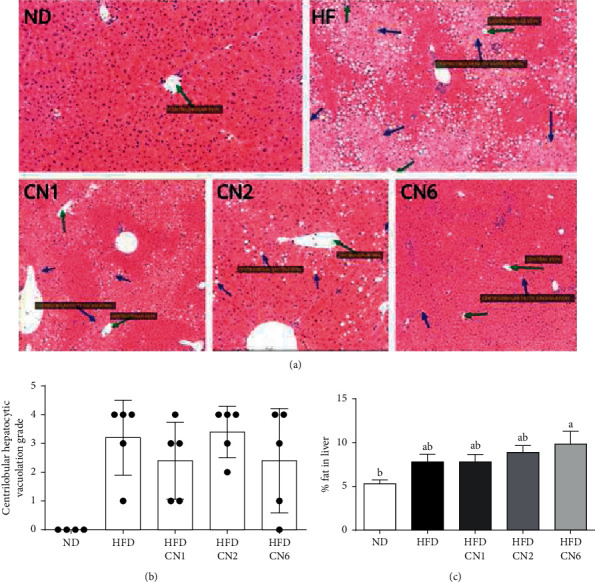
Effect of cannabis extracts on the liver in mice fed with a high-fat diet. (a) Representative liver H&E staining. (b) Steatosis grade. (c) Total lipids in 100 mg liver. All values are expressed as mean ± SEM (*n* = 8–10). Columns marked with different letters (a, b) are significantly different (*p* < 0.05). Histopathological changes were scored by the study pathologist, using semiquantitative grading of four grades (0–4), taking into consideration the severity of the changes (0: no lesion; 1: minimal change; 2: mild change; 3: moderate change; and 4: marked change).

Mice were divided into two groups, which were fed with ND or HFD. Twelve weeks of HFD-regime resulted in a significant increase in BW (Figure 1(a)) despite a decrease in food intake by this group (Figure 1(b)). Increased BW was accompanied by increased insulin resistance as indicated by higher fasting blood glucose levels and reduced glucose tolerance (Figure 1(c)). For the next 8 weeks (weeks 13–20), HFD-fed mice were subdivided into four groups receiving an experimental diet supplemented with extracts from different cannabis varieties (CN1, CN2, or CN6) or vehicle. BW equally increased, while food intake decreased in all HFD-fed groups, regardless of cannabis extract addition (Figures 2(a) and 2(b)). Liver and adipose tissue weight tended to be or were significantly higher in all HFD-fed groups, respectively (Figures 2(c) and 2(d)). Yet, the group in which HFD was supplemented with CN6 extract demonstrated lower adipose tissue weight compared to other HFD-fed groups. To further appraise the influences elicited by cannabis extracts addition to a HFD, additional analyses of liver histology and blood biochemistry were carried out. A similar grade of liver steatosis was disclosed by histology in all HFD-fed groups and was also corroborated biochemically (Figures 3(a)–3(c)). Consistently, blood liver enzymes levels were unaltered by all treatments, including HFD per se (Table 3). Furthermore, histopathological changes were scored (Figure 3(b)) and show a decrease in the mean severity of centrilobular hepatocytic vacuolation, consistent with fatty change, in the CN1- and CN6-treated groups when compared to the HFD alone group.

### 3.2. Blood Lipids Profile and Insulin Sensitivity

Blood total cholesterol and HDL levels increased whereas TG levels did not markedly change by HFD-feeding. No noticeable impact for cannabis extracts was found on blood lipid profile (Table 3). Comparably, as shown in Figure 2(e), impaired insulin sensitivity, evoked by HFD feeding, was correspondingly registered in all HFD groups, as judged by fasting glucose and glucose tolerance at the 19th week of the experiment.

### 3.3. Key Liver Metabolic Pathways/Player and Endocannabinoid Receptors Levels

**Figure 4:**
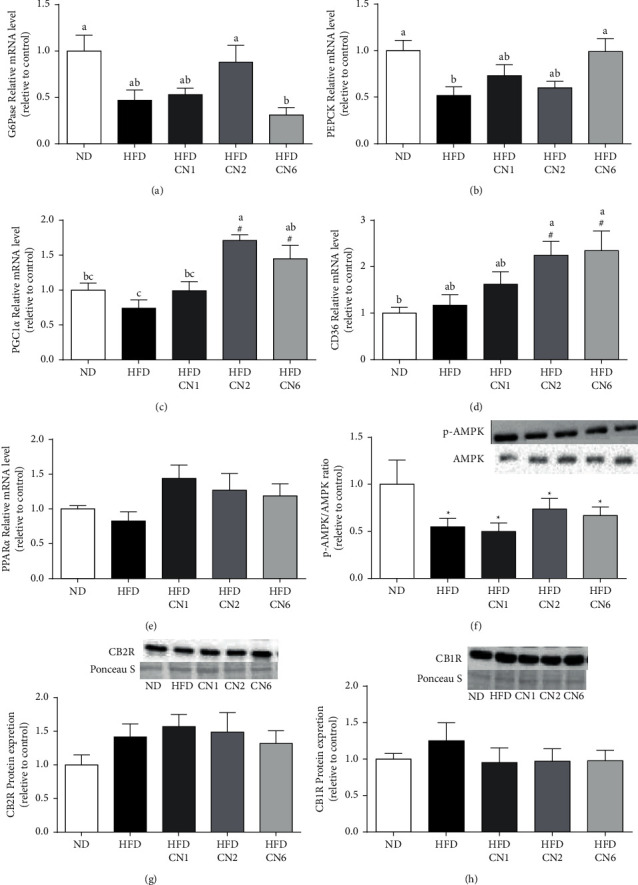
Effect on gluconeogenesis, energy homeostasis, lipid metabolism, and endocannabinoid receptors in the liver. Male C57BL/6 J mice fed high-fat diet supplemented with CN1 or CN2 or CN6 extracts 5 mg/kg BW for 8 weeks. (a) G6pase mRNA levels. (b) PEPCK mRNA levels. (c) CD36 mRNA levels. (d) PGC-1*α* mRNA levels. (e) PPAR*α* mRNA levels. (f) p-AMPK/AMPK protein levels. (g) CB2 receptor protein expression. (h) CB1 receptor protein expression. All values are expressed as mean ± SEM (*n* = 6–8). Columns marked with ^*∗*^ are different from ND (*p* < 0.05). Columns marked with different letters are significantly different (*p* < 0.05).

While HFD decreased gluconeogenic enzymes (G6Pase and PEPCK) gene expression compared with ND, some of the cannabis extracts mitigated this reduction (Figures 4(a) and 4(b)). Interestingly, this was gene dependent, with CN2 increasing only G6Pase expression and CN6 increasing only PEPCK expression. The expression of the co-activator, PGC-1*α*, and the long fatty-acids transporter CD36 were significantly elevated in CN2- and CN6-treated groups (Figures 4(c) and4(d)). In contrast, no difference was observed in the PPAR*α* gene expression between all groups (Figure 4(e)). The fundamental metabolic enzyme, AMPK, which constitutes a central metabolic hub, was downregulated by HFD as depicted by the ratio between phosphorylated AMPK at thr172 in the alpha subunit and total AMPK, a well-established hallmark for its activation. The addition of cannabis extracts did not alleviate this metabolic aberration (Figure 4(f)). Finally, cannabis extracts' addition to a HFD did not profoundly affect the protein contents of CB1 and CB2 receptors of the endocannabinoid system in the liver, although a slight decrease wasobserved in CB1 (Figures 4(g) and 4(h)).

### 3.4. Microbiome Profile

Alterations in microbiota composition and diversity indexes following the treatments were evaluated at all taxonomical levels and are elaborated in Table 4. The observed species index, which reflects the amount of unique species in each group, was lessened in all HFD groups compared to ND group. CN2-added group exhibited an even lower score than that of CN6-added group. Bacterial richness, here represented by community diversity (Shannon index), declined in all HFD-fed groups compared to ND group with the exception of CN6-added group, in which the score was similar to that of the control.

At the phylum level, CN1 extract supplementation resulted in the lowest *Bacteroidetes* abundance and the highest *Firmicutes* abundance compared to all HFD groups. Consequently, *Bacteroidetes/Firmicutes* (B/F) ratio decreased by this treatment. At the class level, *Clostridia* and *Flavobacteria* abundance increased in the HFD + CN1 group compared to all HFD-fed mice. HFD + CN2 group presented lower *Betaproteobacteria* abundance compared to CN6 supplemented group. At the family level, *Deferribacteraceae* abundance increased in CN1 and CN2 groups in comparison to the control group. At the genus level, CN6 extract increased *Bifidobacterium* and *Prevotella* abundance compared to all HFD groups. Sutterella abundance increased in the CN6 group, but was reduced in the CN1 and CN2 groups, though not signifcantly. A marked increase in *Lactobacillus* abundance was observed in the group fed with CN6 extract compared to the HFD group. CN1 treatment elevated *Roseburia* abundance compared to all experimental groups. At the species level, *Akkermansia muciniphila* abundance was higher in CN6 fed group compared to the control.

## 4. Discussion

Though much attention has been pointed to synthetic agonists and antagonists of the endocannabinoid system, the role of naturally derived cannabinoids on the pathogenesis and progression of NAFLD has been less elucidated. The present study estimated the prominent metabolic and microbial modifications emanating from the supplementation of HFD with cannabis plant extract on the progression of NAFLD. Specifically, this work deemed the repercussions of different extraction from three cannabis strains, CN1, CN2, and CN6, which differ in their cannabinoid contents, under this pathologic condition. While CN1 extract is CBD-rich, that of CN2 is THC-rich and CN6 comprises equivocal quantities of these cannabinoids.

No profound alterations were disclosed in typical obesity features nor central metabolic hubs by cannabis extracts addition irrespective of the strain tested. Indeed, BW continued to escalate despite cannabinoids addition. Consist with that, liver and adipose weight tended to increase or significantly increased in all HFD-fed groups with only a minor effect elicited by CN6 extract, concerning adipose tissue weight. Likewise, cannabis extracts did not ameliorate blood lipid profile nor alleviate the abnormalities in glucose metabolism induced by HFD consumption. Concomitantly, gluconeogenesis enzymes expression levels and AMPK activation decreased or tended to decrease in all CN treated groups, albeit, levels did not differ considerably from those of the HFD group, excluding the PEPCK expression of the CN6 group. The only distinguished results are of CD36 and PGC-1alpha in which their mRNA levels in the liver exceeded those of the HFD group. Nevertheless, given the inconsistency with liver steatosis (e.g., the absence of changes in the amounts of fat stored in hepatocytes), it appears that the consequences impendent by these modifications are fairly restricted. Collectively, the findings obtained here argue for the lack of eminent metabolic outcomes provoked by the cannabis extracts tested in this study. However, arguably, this incapacity is plausibly due to the relatively short duration of the study and/or low extract quantity used. It is conjectured longer and/or higher extracts concentrations could have implemented more profound metabolic outcomes. It would be tempting to contend that the absence of modifications in CB receptors reinforces the surmise that treatments fall short to harness this pathway. However, the relationship between phytocannabinoids and NAFLD was shown to be independent of cannabinoid receptors [27], thus suggesting the fact that these receptors or fluctuation in their levels are not imperative for executing effects.

The gut microbiota encompasses a fascinatingly diverse population of microorganisms known to impose mark impacts on the host's health. The interplay between diet pattern and composition, microbiome, and health are attractive subjects in the field of metabolism. A growing body of evidence advocates a profound effect of the gut microbiota in the pathogenesis of obesity and NAFLD [28]. The influential role of the endocannabinoid system on the microbiome has received some attention [29, 30]. Nevertheless, little is known about the repercussion of cannabis and phytocannabinoids specifically on the gut microbiota composition. Accordingly, this work also aimed to delineate whether such modifications exist. At the phylum level, *Bacteroidetes* and *Firmicutes* populations were shown to be the most dominant in the gut [31, 32], with ratios being changed according to the treatment applied. When comparing the microbiota of obese humans and mice to the microbiota of lean, healthy individuals, a lower ratio of B/F is observed [33]. Hence, B/F ratio is customarily utilized as a potential hallmark of the obesity phenotype. Surprisingly, an elevated B/F ratio was observed in all HFD-fed groups, excluding CN1 treated group. This finding is in accord with the recent intense criticism that is directed toward the reliability of this ratio. Indeed, human studies, including one conducted in NAFLD patients, have demonstrated an opposite effect, with HFD increasing rather than decreasing B/F ratio [34, 35]. Therefore, a lower B/F ratio in the CN1 group may argue for the favorable aspects of this extract. What is more, while the abundance of the *Clostridia* class of the *Firmicutes* phylum was substitutionally diminished by HFD feeding, this effect was mitigated by CN1 extract addition. Several works have proposed that elevated *Clostridia* richness may confer protective effects against different kinds of allergens as well as facilitate intestinal integrity by upregulating IL-22 expression. Although not manifested here, elevated *Clostridia* levels may hamper lipid absorption and thus protect from obesity [36–38], Particularly, *Roseburia* spp. was significantly elevated in CN1 treated mice. It was surmised that these short-chain fatty acids- (SCFA) producing bacteria possess anti-inflammatory activities, among other beneficial effects, and may be utilized as a health biomarker or as a probiotic [39]. A discrepancy was revealed in the *Bacteriodetes* phylum of the CN1 group. This phylum is composed of two classes, *Bacteroidia and Flavobacteria*. While the former's levels flourished by the consumption of a HFD, the latter class remained largely identical. Nevertheless, *Bacteroidia* overgrowth was lessened by CN1 addition whereas *Flavobacteria* copiousness was enhanced above that of the other HFD-fed groups. In previous works, *Flavobacterium* presence was hindered by HFD while its enrichment was positively correlated to physical activity [40, 41]. Moreover, it may abolish the development of obesity and NAFLD [41]. Notwithstanding that those outcomes imbued by CN1 extract do appear desired, a noteworthy alteration is an increase in the presence of *Deferribacteraceae*, the *Deferribacteres* phylum and foremost of the *Mucispirillum* genus. Such growth was described in obese or diabetic animals and was implicated as one of the culprits in the pathogenesis of inflammation [42–44].

In comparison with other extracts, CN6 extract supplementation to an HFD triggered the most notable changes in miscellaneous bacterial groups. Several positive changes in gut microbiota have been revealed particularly in this group.Whereas quantities of *Bifidobacterium* and *Lactobacillus* of the *Actinobacteria* and *Bacilli* class, respectively, decreased by HFD, their levels remained intact in the CN6 group. Both genera are well acknowledged for their health-promoting effects, including metabolic disorders and/or antitumor role. Nevertheless, it is important to comprehend that inconsistency exists where diverse species and strains may elicit specific effects [45–51]. Likewise, the CN6-treated mice also attained a substantial increase in the *Prevotella* genus, which was linked to an improvement in glucose metabolism and fermentation potential of complex polysaccharides [52–54].

Comparably, although a propensity toward higher *Akkermansia muciniphila* was observed in all HFD-fed mice, it reached significance merely in the CN6 group. Recent evidence links *Akkermansia muciniphila* with improved metabolic and immunologic functions of the host, including those related to glycemic control and gut immunity [52, 55]. Given the beneficial effects attributed to *Akkermansia muciniphila,* CN6 extract addition to an HFD appears to imbue valuable outcomes, as it naturally accommodates the deployment of these beneficial bacteria. However, as it was disclosed in the CN1 group, not all changes were favorable. Indeed, the levels of *Betaproteobacteria* class of the *Proteobacteia* phylum significantly increased in the CN6 supplemented group and were attributed to the flourishment of *Sutterella* genus. This genus has been suggested to be held responsible for promoting gastrointestinal perturbations. Yet, recent data imply *Sutterella* does not provoke the initiation of inflammation but rather impinge gut immune homeostasis, though this capability is not conserved [48]. Although these findings reveal a significant impact of phytocannabinoids on the microbiota, a clear vision regarding their consequences on the host's health is currently difficult to delineate.

## 5. Conclusions

In summary, in the present work, the addition of different cannabis plant strains extracts of distinct CBD/THC profiles to an HFD unsuccessfully mitigated metabolic perturbations evoked by this diet. Yet, results demonstrated these extracts did establish a considerable impact on the microbiota pattern, which was primarily positive. Further in-depth research is needed to elucidate how diverse cannabis plants affect NAFLD development as well as other metabolic disorders. For this purpose, one must consider the chemical composition of the cannabis strain, the extraction method, doses, given form, and treatment duration.

## Figures and Tables

**Table 1:** Compositions of animal diets.

Ingredients	Normal diet (ND)		High-fat diet (HFD)	
	gr	Kcal	gr	Kcal
Casein	210	840	265	1060
L-Methionine	3	12	4	16
Cornstarch	500	2000	0	0
Dextrose	100	400	160	640
Sucrose	39.15	156.6	90	360
Lard	20	180	310	2790
Soybean oil	20	180	30	270
Anhydrous milk fat	20	180	0	0
Cellulose	35	0	65.5	0
Cholesterol	0	0	0	0
Cholic acid	0	0	0	0
Mineral mix	35	0	51.4	0
Vitamin mix	15	0	21	0
Choline chloride	2.75	0	3	0
BHT	0.014	0	0.014	0
Total energy (kcal)	1000	3769	1000	5136

BHT: butylated hydroxytoluene.

**Table 2:** Sequences of the primers used for quantitative real-time PCR.

Name	Reverse	Forward
18s	5′-CCTCAGTTCCGAAAACCAAC-3′	5′-ACCGCAGCTAGGAATAATGG-3′
PPAR*α*	5′-CTGCGCATGCTCCGTG-3′	5′-CTTCCCAAAGCTCCTTCAAAAA- 3′
CD36	5′-AAAGGCATTGGCTGGAAGAA-3′	5′-TCCTCTGACATTTGCAGGTCTATC-3′
G6pase	5′-AAGAGATGCAGGAGGACCAA-3′	5′-ACTCCAGCATGTACCGGAAG-3′
PEPCK	5′-TGCAGGCACTTGATGAACTC-3′	5′-CAAACCCTGCCATTGTTAAG-3′
PGC-1*α*	5′-AGAGCAAGAAGGCGACACAT-3′	5′-AACAAGCACTTCGGTCATCC-3′

G6pase: glucose 6-phosphatase; PPAR*α*: peroxisome proliferator-activated receptor alpha; CD36: cluster of differentiation 36; PGC-1*α*: peroxisome proliferator-activated receptor gamma coactivator 1-alpha; and PEPCK: phosphoenolpyruvate carboxykinase.

**Table 3:** Serum lipid profile and liver enzymes of mice fed with a high-fat diet supplemented with cannabis extracts.

	ND	HFD	HFD CN1	HFD CN2	HFD CN6
Cholesterol (mg/dl)	104.87 ± 6.13^b^	175.87 ± 12.5^a^	173.1 ± 7.42^a^	193.8 ± 9.97^a^	172.55 ± 17.3^a^
HDL cholesterol (mg/dl)	85.5 ± 5.26^b^	136.8 ± 9.07^a^	132.74 ± 4.09^a^	147.76 ± 5.73^a^	128.27 ± 12.8^a^
Triglycerides (mg/dl)	91.62 ± 4.81	89.4 ± 9.3	83.8 ± 461	90.5 ± 4.9	90.8 ± 15.1
ALP (*μ*l/l)	65.37 ± 3.22	58.57 ± 3.55	55.1 ± 1.77	62.2 ± 4.26	61.55 ± 6.58
AST (*μ*l/l)	93.14 ± 14.01	88.42 ± 10.3	65.5 ± 2.5	81.55 ± 7.96	113.88 ± 28.5
ALT (*μ*l/l)	85.42 ± 21.2	79.42 ± 14.9	53.4 ± 5.57	75.66 ± 11.4	76.62 ± 22.01

All values are expressed as mean ± SEM (*n* = 7–9). Values marked with different letters (a, b) are significantly different (*p* < 0.05).

**Table 4:** Effect of cannabis extracts addition to obese mice during the consumption of an HFD on microbiota composition.

	ND	HFD	HFD + CN1	HFD + CN2	HFD + CN6
*α-Diversity*					
Pielou_e	0.90 ± 0.01	0.89 ± 0.01	0.91 ± 0.01^€^	0.88 ± 0.00^$^	0.91 ± 0.00^#^
Observed OUT's	144.25 ± 2.39	108.40 ± 8.51^*∗*^	106.25 ± 9.75^*∗*^	89 ± 2.47^*∗*^^,$^	116.20 ± 10.09^*∗*^
Shannon index	6.43 ± 0.06	6.00 ± 0.09^*∗*^	6.09 ± 0.10^*∗*^^,€^	5.71 ± 0.05^*∗*^^,$,#^	6.22 ± 0.09

*Phylum level*					
Actinobacteria	0.34 ± 0.17	0.17 ± 0.11	0.30 ± 0.14	0.18 ± 0.15^$^	0.69 ± 0.21^#^
Bacteroidetes	42.50 ± 1.92	67.16 ± 3.44^*∗*^	51.48 ± 7.13^#,$,€^	65.88 ± 3.82^*∗*^	66.49 ± 2.06^*∗*^
Deferribacteres	0.56 ± 0.18	1.81 ± 0.50^*∗*^	3.45 ± 1.26	3.14 ± 0.90^*∗*^	1.86 ± 0.37
Firmicutes	49.78 ± 2.18	21.14 ± 2.82^*∗*^	36.59 ± 6.38^*∗*^^,#,€^	24.83 ± 2.54^*∗*^	23.74 ± 1.01^*∗*^
Fusobacteria	1.65 ± 0.40	0.59 ± 0.25^*∗*^	2.01 ± 0.49^#,€,$^	0.84 ± 0.10	0.66 ± 0.28^*∗*^
Verrucomicrobia	0.18 ± 0.18	0.96 ± 0.10	0.66 ± 0.40	0.90 ± 0.41	1.17 ± 0.41^*∗*^
B/F ratio	0.87 ± 0.08	3.43 ± 0.51^*∗*^	1.75 ± 0.51^#,€,$^	2.85 ± 0.48^*∗*^	2.84 ± 0.22^*∗*^

*Class level*					
Actinobacteria	0.33 ± 0.17	0.10 ± 0.04	0.16 ± 0.06^€,$^	0.15 ± 0.13	0.63 ± 0.19#
Bacteroidia	40.20 ± 1.64	66.40 ± 3.19^*∗*^	48.18 ± 7.88^#,$,€^	64.45 ± 3.80^*∗*^	65.71 ± 2.11^*∗*^
Flavobacteriia	2.30 ± 0.43	0.76 ± 0.44	3.30 ± 0.93^#,$,€^	1.40 ± 0.22	0.78 ± 0.35
Deferribacteres	0.56 ± 0.18	1.81 ± 0.50	3.45 ± 1.26^*∗*^	3.14 ± 0.90^*∗*^	1.86 ± 0.37
Bacilli	2.61 ± 0.54	1.19 ± 0.35^*∗*^	2.35 ± 0.56	1.74 ± 0.36	1.38 ± 0.26
Clostridia	45.94 ± 2.63	18.14 ± 3.10^*∗*^	32.30 ± 6.19^*∗*^^,#,$,€^	21.66 ± 2.22^*∗*^	20.26 ± 0.94^*∗*^
Fusobacteriia	1.65 ± 0.40	0.59 ± 0.25^*∗*^	2.01 ± 0.49^#,$,€^	0.84 ± 0.10	0.66 ± 0.28^*∗*^
Betaproteobacteria	0.46 ± 0.26	1.18 ± 0.45	1.06 ± 0.33	0.83 ± 0.23^$^	1.88 ± 0.43^*∗*^
Verrucomicrobiae	0.18 ± 0.18	0.96 ± 0.10	0.66 ± 0.40	0.90 ± 0.41	1.17 ± 0.41^*∗*^

*Order level*					
Bifidobacteriales	0.33 ± 0.17	0.10 ± 0.04	0.16 ± 0.06^$^	0.15 ± 0.13^$^	0.63 ± 0.19^#^
Bacteroidales	40.20 ± 1.64	66.40 ± 3.19^*∗*^	48.18 ± 7.88^#,$,€^	64.45 ± 3.80^*∗*^	65.71 ± 2.11^*∗*^
Flavobacteriales	2.30 ± 0.43	0.76 ± 0.44	3.30 ± 0.93^#,$,€^	1.40 ± 0.22	0.78 ± 0.35
Deferribacterales	0.56 ± 0.18	1.81 ± 0.50	3.45 ± 1.26^*∗*^	3.14 ± 0.90^*∗*^	1.86 ± 0.37
Bacillales	1.64 ± 0.57	1.06 ± 0.31	2.04 ± 0.48^$^	1.50 ± 0.25	0.72 ± 0.32
Lactobacillales	0.97 ± 0.23	0.13 ± 0.09^*∗*^	0.31 ± 0.11^*∗*^	0.24 ± 0.20^*∗*^	0.66 ± 0.16^#^
Clostridiales	45.94 ± 2.63	18.14 ± 3.10^*∗*^	32.30 ± 6.19^*∗*^^,#,$,€^	21.66 ± 2.22^*∗*^	20.26 ± 0.94^*∗*^
Fusobacteriales	1.65 ± 0.40	0.59 ± 0.25^*∗*^	2.01 ± 0.49^#,$,€^	0.84 ± 0.10	0.66 ± 0.28^*∗*^
RF32	0.02 ± 0.01	0.14 ± 0.05	0.32 ± 0.12	0.25 ± 0.17	0.41 ± 0.20^*∗*^
Burkholderiales	0.44 ± 0.26	1.18 ± 0.45	1.06 ± 0.33	0.83 ± 0.23^$^	1.88 ± 0.43^*∗*^
RF39	0.3 ± 0.07	0.02 ± 0.02^*∗*^	0.00 ± 0.00^*∗*^	0.00 ± 0.00^*∗*^	0.01 ± 0.01^*∗*^
Verrucomicrobiales	0.18 ± 0.18	0.96 ± 0.10	0.66 ± 0.40	0.90 ± 0.41	1.17 ± 0.41^*∗*^

*Family level*					
Bifidobacteriaceae	0.33 ± 0.17	0.10 ± 0.04	0.16 ± 0.06^$^	0.15 ± 0.13^$^	0.63 ± 0.19^#^
Bacteroidaceae	7.48 ± 1.42	43.58 ± 2.42^*∗*^	27.83 ± 5.38^*∗*^^,#,€^	45.35 ± 5.59^*∗*^	37.31 ± 4.24^*∗*^
Prevotellaceae	0.11 ± 0.04	0.21 ± 0.08	0.08 ± 0.04^$^	0.13 ± 0.05^$^	0.61 ± 0.17^*∗*^^,#^
Rikenellaceae	6.96 ± 0.60	3.19 ± 0.39^*∗*^	3.53 ± 1.03^*∗*^	3.63 ± 0.55^*∗*^	5.05 ± 0.86
S24-7	10.98 ± 1.17	8.98 ± 0.95	7.70 ± 1.31^*∗*^	5.87 ± 1.31^*∗*^^,$^	9.87 ± 0.50
Odoribacteraceae	1.65 ± 0.21	1.28 ± 0.44	1.31 ± 0.30	1.69 ± 0.24	2.41 ± 0.56^*∗*^
Flavobacteriaceae	2.30 ± 0.43	0.76 ± 0.44	3.30 ± 0.90^#,$,€^	1.40 ± 0.22	0.78 ± 0.35
Deferribacteraceae	0.56 ± 0.18	1.81 ± 0.5	3.45 ± 1.26^*∗*^	3.14 ± 0.90^*∗*^	1.86 ± 0.37
Bacillaceae	1.63 ± 0.57	1.06 ± 0.31	2.02 ± 0.48^$^	1.50 ± 0.25	0.72 ± 0.32
Lactobacillaceae	0.97 ± 0.23	0.13 ± 0.09^*∗*^	0.31 ± 0.11^*∗*^	0.23 ± 0.20^*∗*^	0.66 ± 0.16^#^
Christensenellaceae	0.75 ± 0.11	0.28 ± 0.08^*∗*^	0.39 ± 0.13^*∗*^	0.31 ± 0.13^*∗*^	0.29 ± 0.05^*∗*^
Clostridiaceae	0.94 ± 0.07	0.29 ± 0.19^*∗*^	0.46 ± 0.21	0.52 ± 0.24	0.28 ± 0.09^*∗*^
Dehalobacteriaceae	0.54 ± 0.07	0.63 ± 0.11	0.87 ± 0.17^*∗*^^,$,€^	0.45 ± 0.08	0.38 ± 0.08
Lachnospiraceae	5.87 ± 1.35	4.00 ± 0.54	7.66 ± 1.10^#,€^	3.05 ± 0.60^*∗*^	5.21 ± 0.45
Peptococcaceae	0.69 ± 0.12	0.11 ± 0.08^*∗*^	0.24 ± 0.15^*∗*^	0.06 ± 0.03^*∗*^	0.35 ± 0.21
Ruminococcaceae	18.76 ± 1.92	7.30 ± 1.23^*∗*^	10.80 ± 2.25^*∗*^^,$^	10.15 ± 0.56^*∗*^	6.21 ± 0.35^*∗*^
Fusobacteriaceae	1.65 ± 0.40	0.59 ± 0.25^*∗*^	2.01 ± 0.49^#,$,€^	0.84 ± 0.10	0.66 ± 0.28^*∗*^
Alcaligenaceae	0.37 ± 0.28	1.14 ± 0.43	0.85 ± 0.34	0.80 ± 0.22^$^	1.88 ± 0.43^*∗*^
Verrucomicrobiaceae	0.18 ± 0.18	0.96 ± 0.10	0.66 ± 0.40	0.90 ± 0.41	1.17 ± 0.41^*∗*^

*Genus level*					
*Bifidobacterium*	0.33 ± 0.17	0.10 ± 0.04	0.16 ± 0.06^$^	0.15 ± 0.13^$^	0.63 ± 0.19^#^
*Bacteroides*	7.48 ± 1.42	43.58 ± 2.42^*∗*^	27.83 ± 5.38^*∗*^^,#,€^	45.35 ± 5.59^*∗*^	37.31 ± 4.24^*∗*^
*Prevotella*	0.11 ± 0.04	0.21 ± 0.08	0.08 ± 0.04^$^	0.13 ± 0.05^$^	0.61 ± 0.17^*∗*^^,#^
*Butyricimonas*	0.55 ± 0.17	1.06 ± 0.39	0.89 ± 0.25	1.37 ± 0.28	1.87 ± 0.49^*∗*^
*Odoribacter*	1.10 ± 0.37	0.22 ± 0.10^*∗*^	0.43 ± 0.22	0.32 ± 0.22^*∗*^	0.54 ± 0.20
*Mucispirillum*	0.56 ± 0.18	1.81 ± 0.50	3.45 ± 1.26^*∗*^	3.14 ± 0.90^*∗*^	1.86 ± 0.37
*Lactobacillus*	0.97 ± 0.23	0.13 ± 0.09^*∗*^	0.31 ± 0.11^*∗*^	0.23 ± 0.20^*∗*^	0.66 ± 0.16^#^
*Dehalobacterium*	0.54 ± 0.07	0.63 ± 0.11	0.87 ± 0.17^*∗*^^,$,€^	0.45 ± 0.08	0.38 ± 0.08
*Coprococcus*	0.70 ± 0.21	0.21 ± 0.12^*∗*^	0.31 ± 0.13^*∗*^	0.20 ± 0.06^*∗*^	0.22 ± 0.06^*∗*^
*Roseburia*	0.00 ± 0.00	0.08 ± 0.08	1.33 ± 0.72^*∗*^^,#,€^	0.09 ± 0.09	0.63 ± 0.11
*Oscillospira*	6.37 ± 0.70	1.86 ± 0.41^*∗*^	3.58 ± 1.33^*∗*^	3.71 ± 0.37^*∗*^	1.72 ± 0.16^*∗*^
*Ruminococcus*	1.14 ± 0.40	0.42 ± 0.20	1.12 ± 0.33	1.43 ± 0.23^#,$^	0.52 ± 0.22
*Cetobacterium*	1.65 ± 0.40	0.59 ± 0.25^*∗*^	2.01 ± 0.49^#,$,€^	0.84 ± 0.10	0.66 ± 0.28^*∗*^
*Sutterella*	0.37 ± 0.28	1.14 ± 0.43	0.83 ± 0.35^$^	0.80 ± 0.22^$^	1.88 ± 0.43^*∗*^
*Akkermansia*	0.18 ± 0.18	0.96 ± 0.10	0.66 ± 0.40	0.90 ± 0.41	1.17 ± 0.41^*∗*^

*Species level*					
*distasonis*	0.00 ± 0.00	2.91 ± 0.43	1.96 ± 1.04	1.54 ± 1.54^#^	1.80 ± 0.51
*schaedleri*	0.56 ± 0.18	1.81 ± 0.50	3.45 ± 1.26^*∗*^	3.14 ± 0.90^*∗*^	1.86 ± 0.37
*gnavus*	1.27 ± 0.46	0.84 ± 0.30	1.87 ± 0.51^€^	0.51 ± 0.31	1.12 ± 0.20
*flavefaciens*	0.47 ± 0.23	0.00 ± 0.00^*∗*^	0.00 ± 0.00^*∗*^	0.00 ± 0.00^*∗*^	0.00 ± 0.00^*∗*^
*somerae*	1.65 ± 0.40	0.59 ± 0.25^*∗*^	2.01 ± 0.49^#,$,€^	0.84 ± 0.10	0.66 ± 0.28^*∗*^
*muciniphila*	0.18 ± 0.18	0.96 ± 0.10	0.66 ± 0.40	0.90 ± 0.41	1.17 ± 0.41^*∗*^

The effect of diets on gut microbiota richness and diversity as well as microbiota composition at all taxonomic levels. Male C57BL/6 J mice were fed with a high-fat diet supplemented with CN1 or CN2 or CN6 extracts 5 mg/kg BW for 8 weeks. Values are expressed as mean ± SEM (*n* = 5). ^*∗*^*p* < 0.05 versus ND group; ^#^*p* < 0.05 versus HFD; ^$^*p* < 0.05 versus CN6 group; and ^€^*p* < 0.05 versus CN2 group.

## Data Availability

All raw sequence data were uploaded to NCBI Sequence Read Archive (SRA) under BioProject accession no. PRJNA809153.

## Abbreviations

ALT: Alanine aminotransferase
ALP: Alkaline phosphatase
AST: Aspartate aminotransferase
BW: Body weight
CBD: Cannabidiol
HDL: High-density lipoprotein
H&E: Hematoxylin and eosin
HFD: High-fat diet
NAFLD: Nonalcoholic fatty liver disease
ND: Normal diet
OGTT: Oral glucose tolerance test
THC: Delta-9-tetrahydrocannabinol
TG: Triglycerides.
